# Supplementary material for: Secondary Metabolite Variation and Bioactivities of Two Marine Aspergillus Strains in Static Co-Culture Investigated by Molecular Network Analysis and Multiple Database Mining Based on LC-PDA-MS/MS
Source: Antibiotics (Basel). 2022 Apr 12;11(4):513. doi: 10.3390/antibiotics11040513 (PMC9031932; doi:10.3390/antibiotics11040513)
Supplement: Supplementary file 1 [file antibiotics-11-00513-s001.zip › Tables S1 and S2.pdf]

Supporting information

Table S1. The table shows the variation of yields presented by peak areas in extracted ion chromatographies for different compounds

Feature peak number	retention time (min)	Measured m/z	Sample number	Peak Area	Peak Area (G1)	Peak Area (G2)	Fold of change relative to G1	Fold of change relative to G2
1	1.67	309.22	G3	3620879.16		26648277.63		0.14
			G4	91154.56				0.003
			G5	593781.99				0.02
			G6	3522067.80				0.13
			G7	42188129.38				1.58
2	2.87	679.59	G3	76840701.24	16795830.12	48463830.52	4.57	1.59
			G4	95111091.56			5.66	1.96
			G5	102023265			6.07	2.11
			G6	108378583.96			6.45	2.24
			G7	119161695.9			7.09	2.46
3	6.40	777.38	G3	1036727.8	20606542.49		0.05	
			G4	71272594.05			3.46	
			G5	63453608.16			3.08	
			G6	63582.77			0.003	
			G7	212056.87			0.01	
4	7.81	759.36	G3	1134190770	310976824.6		3.65	
			G4	1080810291			3.48	
			G5	241975956.4			0.78	
			G6	28846488.59			0.09	
			G7	42799803.06			0.14	
5	9.30	585.27	G3	3378519.33		14854340.28		0.23
			G4	224474.64				0.02
			G5	1153568.02				0.08
			G6	22146070.5				1.49
			G7	51847648.84				3.49
6	9.99	527.22	G3	8962482.13		74552422.41		0.12
			G4	564095.35				0.008
			G5	8183279.55				0.11
			G6	52275649.97				0.70
			G7	53901881.61				0.72
7	11.80	427.26	G3	4847851.665		22709063.8		0.21
			G4	1764481.85				0.08
			G5	81082898.13				3.57
			G6	71445604.45				3.15
			G7	100354050.6				4.42
8	12.15	459.31	G3	4708411.63		9102325.84		0.52
			G4	1764481.85				0.19

Table S1. Cont.

Feature peak number	Representative retention time (min)	Measured m/z	Sample number	Peak Area	Peak Area (G1)	Peak Area (G2)	Fold of change relative to G1	Fold of change relative to G2
8	12.15	459.31	G5	76557496.58		9102325.84		8.41
			G6	66539257.66				7.31
			G7	95320100.59				10.47
9	16.26	507.38	G3	1297253.48		81381822.13		0.02
			G4	4669779.11				0.06
			G5	235174.36				0.003
			G6	6355606.81				0.08
			G7	4378939.5				0.05
10	17.78	1106.49	G3	114851.45		114851448.1		0.001
			G4	344554.34				0.003
			G5	10336630.33				0.09
			G6	73504926.81				0.64
			G7	156197969.5				1.36
11	18.20	1135.35	G3	701798.51		35089925.51		0.02
			G4	1403597.02				0.04
			G5	18246761.27				0.52
			G6	46669600.93				1.33
			G7	106322474.3				3.03
12	2.16	606.07	G3	895722.73		4478613.63		0.2
			G4	268716.82				0.06
			G5	358289.09				0.08
			G6	89572.27				0.02
			G7	2552809.77				0.57
13	8.87	362.92	G3	21748.05		724935.03		0.03
			G4	14498.70				0.02
			G5	101490.90				0.14
			G6	195732.46				0.27
			G7	659690.88				0.91
14	11.54	367.19	G3	0	0		0	
			G4	0			0	
			G5	47459.20			1 (/ G5)	
			G6	635478.69			13.39 (/ G5)	
			G7	89697.89			1.89 (/ G5)	
15	13.50	441.07	G3	987377.37	4292945.09			0.23
			G4	2404049.25				0.56
			G5	2146472.55				0.5
			G6	601012.31				0.14
			G7	0				0

Table S2: Multiple database mining for the peaks without remarkable yield changes in LC-MS traces between axenic and co-cultures while annotated by GNPS matching

Sample #	m/z measured	Mean retention time (min)	UV maximum measured (nm)	Compound name	Molecular weight in Libraries	Library & ID	MS^2 similarity (cosine)	Molecular Formula	UV maximum in libraries/ literature (nm)	Bioresource	DOI	Biological activity	SMILES code
G3、G5、G6、G7	261.15 [M+H]+	2.38	231	cyclo-(Leu-Phe)	260.32	GNPS,CCMSLIB00000081165	0.86	$C_{15}H_{20}N_2O_2$	260,204,169,141,113,91	*Streptomyces albulus KO-23.*	10.7164/antibiotics.53.58	N/A	O=C(NC1CC(C)C)C(CC2=CC=CC=C2)NC1=O
				cyclo-(Leu-Phe);3-benzyl-6-(2-methylpropyl)piperazine-2,5-dione	260.32	Pubchem，562691	N/A	$C_{15}H_{20}N_2O_2$	260,204,169,141,113,91	*Streptomyces albulus KO-23.*	10.7164/antibiotics.53.58	N/A	CC(C)CC1C(=O)NC(C(=O)N1)CC2=CC=CC=C2
				Aspiperidine oxide	260.33	Dictionary of Natural Products	N/A	$C_{15}H_{20}N_2O_2$	273	*Aspergillus indologenus*	10.1016/j.tetlet.2015.02.082	no biological activity was found.	C[N+]1(CCCCC1C(=O)NC=CC2=CC=CC=C2)[O-]
				Biotin S-Oxide	260.31		N/A	$C_{10}H_{16}N_2O_4S$	N/A	N/A	N/A	N/A	C1[C@H]2[C@@H]([C@@H](S1=O)CCCCC(=O)O)NC(=O)N2
				Cyclo(4-hydroxyprolylphenylalanyl)	260.29		N/A	$C_{14}H_{16}N_2O_3$	N/A(large conjugative system)	*Menella kanisa*	10.1080/14786419.2013.879134	brine shrimp lethality	O=C(N1)[C@H]2N(C[C@H](O)C2)C([C@@H]1CC3=CC=CC=C3)=O
				methyl 2-(6-bromo-3,4-dihydroxyphenyl)acetate	261.07	Natural Product Atlas, NPA000945	N/A	$C_9H_9BrO_4$	290	*Aspergillus* sp.	10.1248/cpb.c12-01048	significant radical scavenging activity against DPPH	COC(=O)CC1=CC(=C(C=C1Br)O)O
G5、G6	227.16 [M+H]+	2.29	232	cyclo-[L-(4-hydroxy-Pro)-L-leu]	226.14	GNPS,CCMSLIB00000081185	0.86	N/A	N/A	*Acremonium* sp.	10.1155/2021/9930210	N/A	N/A
				Euxanthone；1,7-Dihydroxyxanthone	228.2	Pubchem，5281631	N/A	$C_{13}H_8O_4$	N/A(large conjugative system)	*Aspergillus unguis*	10.3109/13880209.2010.494673	radical scavenging activity against DPPH, prevented the growth of Mycobacterium smegmatis, active on M. tuberculosis.	C1=CC(=C2C(=C1)OC3=C(C2=O)C=C(C=C3)O)O
				Agglomerin F	226.18	Dictionary of Natural Products，	N/A	$C_{10}H_{10}O_6$	248,280,314	*Aspergillus niger*	10.1002/cbic.201402172	N/A	COC(=O)CCC(=O)C1=C(C(=C)OC1=O)O
				Csypyrones B1	226.18		N/A	$C_{10}H_{10}O_6$	309,224	*Aspergillus oryzae*	10.1016/j.bmc.2010.04.058	N/A	O=C1C(C(C)=O)=C(O)C=C(CCC(O)=O)O1
				Aspertamarinolide C	226.27	Natural Product Atlas, NPA032140	N/A	$C_{12}H_{18}O_4$	conjugative double bond	*Aspergillus tamarii PSU-MF90*	10.1016/j.tetlet.2020.152529	inactive in all bioassays.	CCCC[C@H]1C[C@@H](C(=O)O1)C(=C)C(=O)OC
G2	405.05 [M+H]+	11.48	240,247,272	lovastatin	404.27	GNPS, CCMSLIB00000839194	0.80	$C_{24}H_{36}O_5$	238	*Aspergillus terreus*	10.1016/j.fct.2019.110585<br>10.1080/10826068.2020.1805624.	action in reducing blood lipid concentration, prevention of cancer and its neuroprotective, anti-inflammatory and antibacterial properties	O=C([C@H](CC)C)O[C@@H]1[C@H]2C(C=C[C@H](C)[C@@H]2CC[C@H]3OC(C[C@@H](C3)O)=O)=C[C@@H](C1)C
				7-bromounguinol	405.24	PubChem,146684023	N/A	$C_{19}H_{17}BrO_5$	201,219,262,323	*Aspergillus unguis*	10.1039/c8ob00545a	activity against the Gram-positive bacteria and reasonable selectivity over mammalian cells	C/C=C(\C)/C1=C(C(=C(C2=C1OC3=C(C(=CC(=C3)O)C)C(=O)O2)C)O)Br
				7-bromounguinol	405.24	Natural Product Atlas, NPA027773	N/A	$C_{19}H_{17}BrO_5$	201,219,262,323	*Aspergillus unguis*	10.1039/c8ob00545a	activity against the Gram-positive bacteria and reasonable selectivity over mammalian cells	C/C=C(\C)/C1=C(C(=C(C2=C1OC3=C(C(=CC(=C3)O)C)C(=O)O2)C)O)Br

Table S2. Cont.

Sample #	m/z measured	Mean retention time (min)	UV maximum measured (nm)	Compound name	Molecular weight in Libraries	Library & ID	MS^2 similarity (cosine)	Molecular Formula	UV maximum in libraries/ literature (nm)	Bioresource	DOI	Biological activity	SMILES code
G6、G7	487.29 [M+Na+	11.74	247,276,313,366	T-2 Toxin	466.50	GNPS,CCMSLIB00004681488	0.74	$C_{24}H_{34}O_9$	weak UV absorption	*Fusarium*	10.1021/jf200767q	Acute toxicity	CC1=CC2C(CC1OC(=O)CC(C)C)(C3(C(C(C(C34CO4)O2)O)OC(=O)C)C)COC(=O)C
				(1'S)-6-O-methyl-7-bromoaverantin	465.29	PubChem, 10458149	N/A	$C_{21}H_{21}BrO_7$	224,289,320,445	*Aspergillus sp. SCSIO F063*	10.1021/np3002699	no biological activity was found	CCCCC[C@@H](C1=C(C=C2C(=C1O)C(=O)C3=C(C(=C(C=C3C2=O)OC)Br)O)O)O
				Arisugacin E; 12aα-Hydroxy, 1,2-didehydro, 3-ketone	466.56	Dictionary of Natural Products	N/A	$C_{27}H_{30}O_7$	208,247,331	*Penicillium* sp.	N/A	N/A	N/A
				17-epi-notoamide M	465.55	Natural Product Atlas, NPA009865	N/A	$C_{26}H_{31}N_3O_5$	231,250,278	Aspergillus sp.	10.1021/np300707x	showed weak or no activity against these pathogenic bacteria, revealed selectivity toward S. *epidermidis*	CC1(C=CC2=C(O1)C=CC3=C2NC(=O)[C@@]3(C[C@H]4C(=O)N5CCC[C@@]5(C(=O)N4)O)C(C)(C)C=C)C
G2、G3、G6	432.18 [M+H]+	8.14	231,304	Isochromophilone VI	434.17	GNPS,CCMSLIB00005722749	0.76	$C_{23}H_{28}ClNO_5$	235,345,370,485	*penicillium multicolor FO-3216*	10.7164/antibiotics.48.696	Antimicrobial and cytotoxic B-16 melanoma cells in vitro activities	CC[C@H](C)\\C=C(C)\\C=C\\C1=CC2=C(Cl)C(=O)[C@](C)(OC(C)=O)C(=O)C2=CN1CCO
				6_,9_-Dihydroxy-14-p-nitrobenzoylcinnamolide	431.43	Pubchem，101891076	N/A	$C_{22}H_{25}NO_8$	254	*Aspergillus ochraceus Jcma1F17*	10.1039/c3md00371j	antiviral activities against H3N2 and EV71	C[C@@]1(CCC[C@]2([C@H]1[C@@H](C=C3[C@@]2(COC3=O)O)O)C)COC(=O)C4=CC=C(C=C4)[N+](=O)[O-]
				Pseurotin A	431.43	Dictionary of Natural Products，	N/A	$C_{22}H_{25}NO_8$	202,251,284	*Aspergillus* sp.	10.1038/ja.2008.99	induction of cell differentiation , inhibition of chitin synthase , inhibition of monoamine oxidase , and apomorphine-antagonistic activity	CC/C=C\[C@@H]([C@@H](C1=C(C(=O)[C@@]2(O1)[C@H]([C@@](NC2=O)(C(=O)C3=CC=CC=C3)OC)O)C)O)O
				6_,9_-dihydroxy-14-p-nitrobenzoylcinnamolide	431.43	Natural Product Atlas, NPA029851	N/A	$C_{22}H_{25}NO_8$	*254*	*Aspergillus ochraceus Jcma1F17*	10.1039/c3md00371j	antiviral activities against H3N2 and EV71	C[C@@]1(CCC[C@]2([C@H]1[C@@H](C=C3[C@@]2(COC3=O)O)O)C)COC(=O)C4=CC=C(C=C4)[N+](=O)[O-]
G1、G2、G3、G4、G5、G6、G7	501.09 [M+Na]+	13.14	273,304,316,342	Butanoic acid, 3-methyl-, 4-[(beta-D-glucopyranosyloxy)methyl]-1,4a,5,6,7,7a-hexahydro-6,7-dihydroxy-7-(hydroxymethyl)cyclopenta[c]pyran-1-yl ester	478.50	GNPS,CCMSLIB00000850710	0.83	$C_{21}H_{34}O_{12}$	N/A	N/A	N/A	N/A	CC(C)CC(=O)OC1C2C(CC(C2(CO)O)O)C(=CO1)CO[C@H]3[C@@H]([C@H]([C@@H]([C@H](O3)CO)O)O)O
				Fumitremorgin B	479.57	Pubchem，105113	N/A	$C_{27}H_{33}N_3O_5$	226,278,295	*Aspergillus fumigatus*	10.1039/C39740000408	N/A	CC(=CCN1C2=C(C=CC(=C2)OC)C3=C1[C@@H](N4C(=O)[C@@H]5CCCN5C(=O)[C@@]4([C@H]3O)O)C=C(C)C)C
				Asnovolin C	478.62	Dictionary of Natural Products，	N/A	$C_{27}H_{42}O_7$	270	*Aspergillus novof umigatus*	10.1021/acs.jnatprod.6b00013	fibronectin expres-sion regulatory factors	C[C@H]1CC[C@H]([C@]([C@]12C[C@@]3([C@H]([C@@H](C(=O)C(=C3O2)C)C(=O)OC)C)C)(C)CCC(=O)OC)C(C)(C)O
				Fumitremorgin B	477.57		N/A	$C_{27}H_{31}N_3O_5$	226,278,295	*Aspergillus fumigatus*	10.1039/C39740000408	N/A	CC(=CCN1C2=C(C=CC(=C2)OC)C3=C1[C@@H](N4C(=O)[C@@H]5CCCN5C(=O)[C@@]4([C@H]3O)O)C=C(C)C)C

Table S2. Cont.

Sample #	m/z measured	Mean retention time (min)	UV maximum measured (nm)	Compound name	Molecular weight in Libraries	Library & ID	MS^2 similarity (cosine)	Molecular Formula	UV maximum in libraries/ literature (nm)	Bioresource	DOI	Biological activity	SMILES code
				29-N-demethylparaherquamide J	477.56	Natural Product Atlas, NPA012534	N/A	$C_{27}H_{31}N_3O_5$	225	*Aspergillus duricaulis*	10.1021/acs.jnatprod.5b00508	N/A	C[C@@H]1C[C@@]23C[C@@H]4[C@@](C[C@@]5(C4(C)C)C6=C(C7=C(C=C6)OC(C=CO7)(C)C)NC5=O)(CN2C1=O)NC3=O
G2、G5、G7	617.41 [M+H]+	14.22	264,342,359	Heme B / Porphyrin	616.49	GNPS,CCMSLIB00000072119	0.82	$C_{34}H_{32}FeN_4O_4$	N/A(large conjugative system)	N/A	10.1073/pnas.0404349101	inhibits Aβ aggregation in vitro and in vivo	CC1=C(C2=CC3=C(C(=C([N-]3)C=C4C(=C(C(=N4)C=C5C(=C(C(=N5)C=C1[N-]2)C)C=C)C)C=C)C)CCC(=O)O)CCC(=O)O.[Fe+2]
				Phomoxanthone D	616.57	Dictionary of Natural Products，	N/A	$C_{30}H_{32}O_{14}$	203,254,374	*Phomopsis* sp. xy21	10.3390/md14110215.	No obvious inhibitory activity	C[C@H]1C[C@@H]([C@@]2(C(=O)C3=C(C=CC(=C3O)C4=C(C5=C(C=C4)O[C@]67CO[C@]([C@@]6(C5=O)O)(C[C@@H]([C@@H]7O)C)O)O)O[C@]2([C@H]1O)CO)O)O
				19-hydroxypenitrem E	615.76	Natural Product Atlas, NPA013252	N/A	$C_{37}H_{45}NO_7$	231,284	*Aspergillus nidulans EN-330*	10.1016/j.phytol.2015.03.017	exhibited cytotoxic activity against brine shrimp	CC(=C)[C@@H]1[C@@H]([C@@H]2[C@@]3(O2)[C@@H](O1)CC[C@]4([C@]3(CC[C@@]5([C@@]4(C6=C7[C@H]5OC([C@H]8C[C@H]9[C@@]8(C1=C(CC9=C)C=CC(=C71)N6)O)(C)C)C)O)O)C)O
G4	755.37 [M+Na]+	3.93	230	Cyclo(glycyltryptophylprolylglycylvalylglycyl-beta-hydroxytyrosyl)	732.31	GNPS,CCMSLIB00000855727	0.77	$C_{36}H_{44}N_8O_9$	N/A	N/A	N/A	N/A	CC(C)C1NC(=O)CNC(=O)C2CCCN2C(=O)C(CC3=C[NH]C4=CC=CC=C34)NC(=O)CNC(=O)C(NC(=O)CNC1=O)C(O)C5=CC=C(O)C=C5
				Talapolyester F	732.73	Natural Product Atlas, NPA008801	N/A	$C_{36}H_{44}O_{16}$	N/A(large conjugative system)	*Talaromyces flavus.*	10.1016/j.tet.2014.02.060	N/A	CC(C)C[C@H]1C(=O)N2CCC[C@H]2C(=O)N[C@H](C(=O)N([C@H](C(=O)N([C@H](C(=O)NCCCCC(=O)O1)CC3=CC=CC=C3)C)C(C)C)C)CC4=CC=CC=C4
G2、G3、G7	478.23 [M+H_2O]+	10.83	255,273,322	4-[5-[[4-[5-[acetyl(hydroxy)amino]pentylamino]-4-oxobutanoyl]-hydroxyamino]pentylamino]-4-oxobutanoic acid	460.50	GNPS,CCMSLIB00000845585	0.75	$C_{20}H_{36}N_4O_8$	N/A	N/A	N/A	N/A	CC(=O)N(O)CCCCCNC(=O)CCC(=O)N(O)CCCCCNC(=O)CCC(O)=O
				Asnovolin D; 4-Deoxy, 4,14-didehydro, Me ester	464.62	Dictionary of Natural Products，	N/A	$C_{26}H_{40}O_7$	*270*	*Aspergillus novofumigatus*	10.1021/acs.jnatprod.6b00013	N/A	C[C@H]1CC[C@H]([C@]([C@]12C[C@@]3([C@H]([C@@H](C(=O)C(=C3O2)C)C(=O)OC)C)C)(C)CCC(=O)O)C(C)(C)O
				Aspergiolide A	460.39		N/A	$C_{25}H_{16}O_9$	203,235,307,431	*Aspergillus glaucus*	10.1016/j.tet.2006.11.074	selective cytotoxicities against A-549, HL-60, BEL-7402, and P388 cell lines.	CC1=CC(=CC(=C1C(=O)C2=C3C4=C(C(=CC(=C4)O)O)C(=O)C5=C(C=C(C(=C35)OC2=O)C)O)O)O
				7-bromofolipastatin	459.33	Natural Product Atlas, NPA012534	N/A	$C_{27}H_{31}N_3O_5$	190,281	*Aspergillus unguis*	10.1039/c8ob00545a	antimicrobial activity against *Bacillus subtilis (ATCC 6633); Staphylococcus aureus (ATCC 25923); Saccharomyces cerevisiae (ATCC 9763); NS-1 (ATCC TIB-18)*	CC=C(C)C1=CC(O)=C(C)C2=C1C(=O)OC1=C(O2)C(C(C)=CC)=C(Br)C(O)=C1C
G5、G6、G7	445.34 [M+H]+	10.56	252,280	Lovastatin Sodium	444.5	GNPS,CCMSLIB00000847300	0.70	$C_{24}H_{37}NaO_6$	N/A	*Monascus.*	10.1016/j.fct.2019.110585	action in reducing blood lipid concentration, prevention of	CC[C@H](C)C(=O)O[C@H]1C[C@H](C=C2[C@H]1[C@H]([C@H](C=C2)C)CC[C@H](C[C@H](CC(=O)[O-])O)O)C.[Na+]

Table S2. Cont.

Sample #	m/z measured	Mean retention time (min)	UV maximum measured (nm)	Compound name	Molecular weight in Libraries	Library & ID	MS^2 similarity (cosine)	Molecular Formula	UV maximum in libraries/ literature (nm)	Bioresource	DOI	Biological activity	SMILES code
												cancer and its neuroprotective, anti-inflammatory and antibacterial properties	
				Fumiquinazoline A	445.50	Pubchem	N/A	$C_{24}H_{23}N_5O_4$	N/A	*Aspergillus* sp.	10.1016/j.jiac.2015.01.005	No antibacterial activity	C[C@H]1C2=NC3=CC=CC=C3C(=O)N2[C@@H](C(=O)N1)C[C@]4([C@H]5N[C@H](C(=O)N5C6=CC=CC=C64)C)O
				Andibenin A	444.52	Dictionary of Natural Products，	N/A	$C_{25}H_{32}O_7$	end absorption	*Aspergillus variecolor*	10.1039/P19790002118	N/A	C[C@@]12C[C@H]3[C@@]([C@]4(CC[C@@]35[C@@]16COC(=O)[C@@H]6[C@@H]([C@](C5)(C2=O)C)O)C=CC(=O)OC4(C)C)(C)O
