# Supplementary figures and images for: Secondary Metabolite Variation and Bioactivities of Two Marine Aspergillus Strains in Static Co-Culture Investigated by Molecular Network Analysis and Multiple Database Mining Based on LC-PDA-MS/MS
Source: Antibiotics (Basel). 2022 Apr 12;11(4):513. doi: 10.3390/antibiotics11040513 (PMC9031932; doi:10.3390/antibiotics11040513)

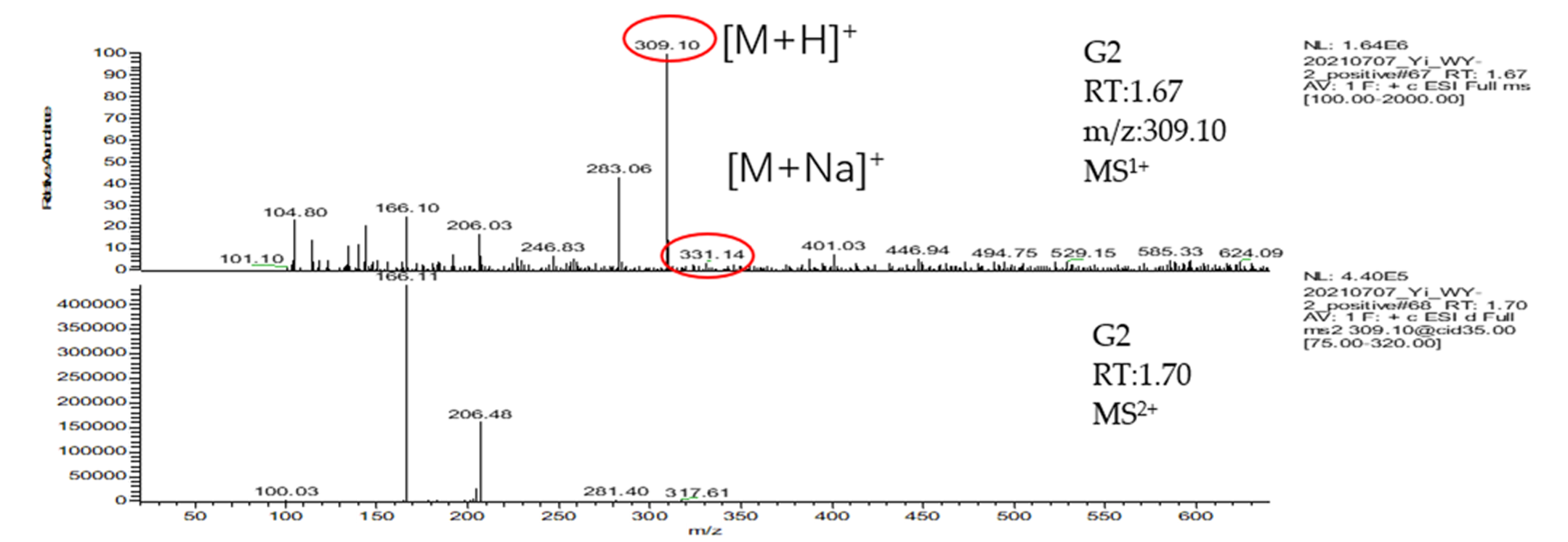

Supplement: Supplementary file 1 [file antibiotics-11-00513-s001.zip › Figure S1.png]

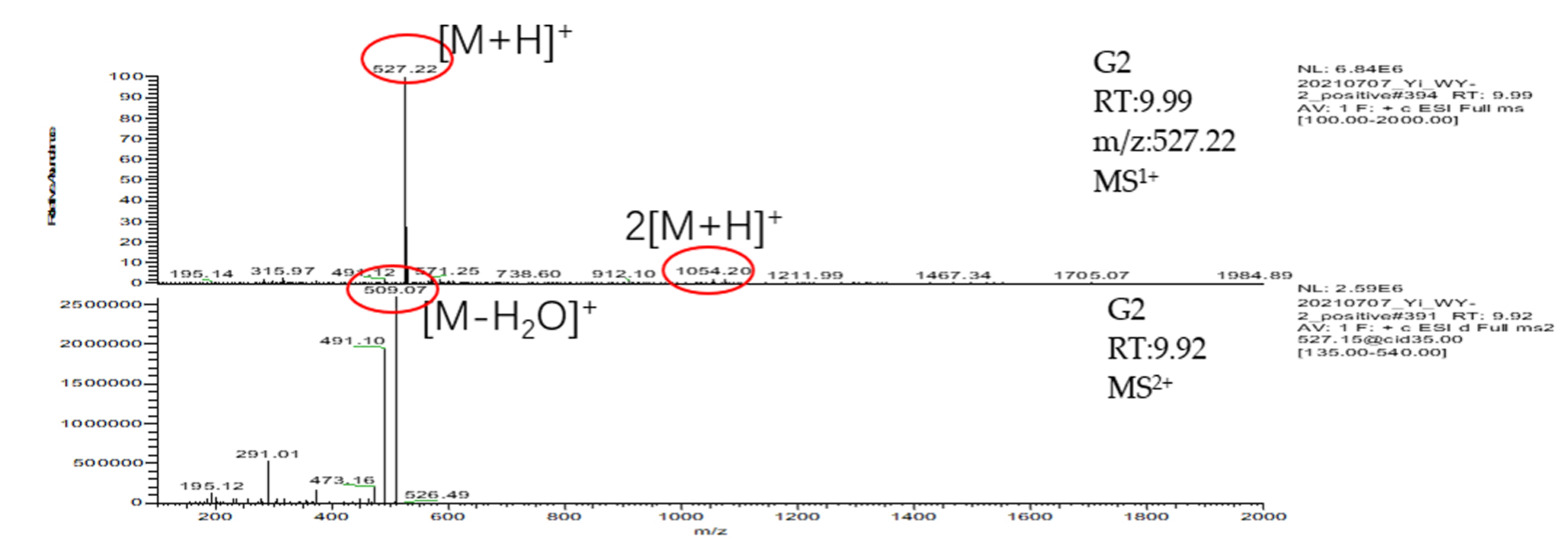

Supplement: Supplementary file 1 [file antibiotics-11-00513-s001.zip › Figure S10.png]

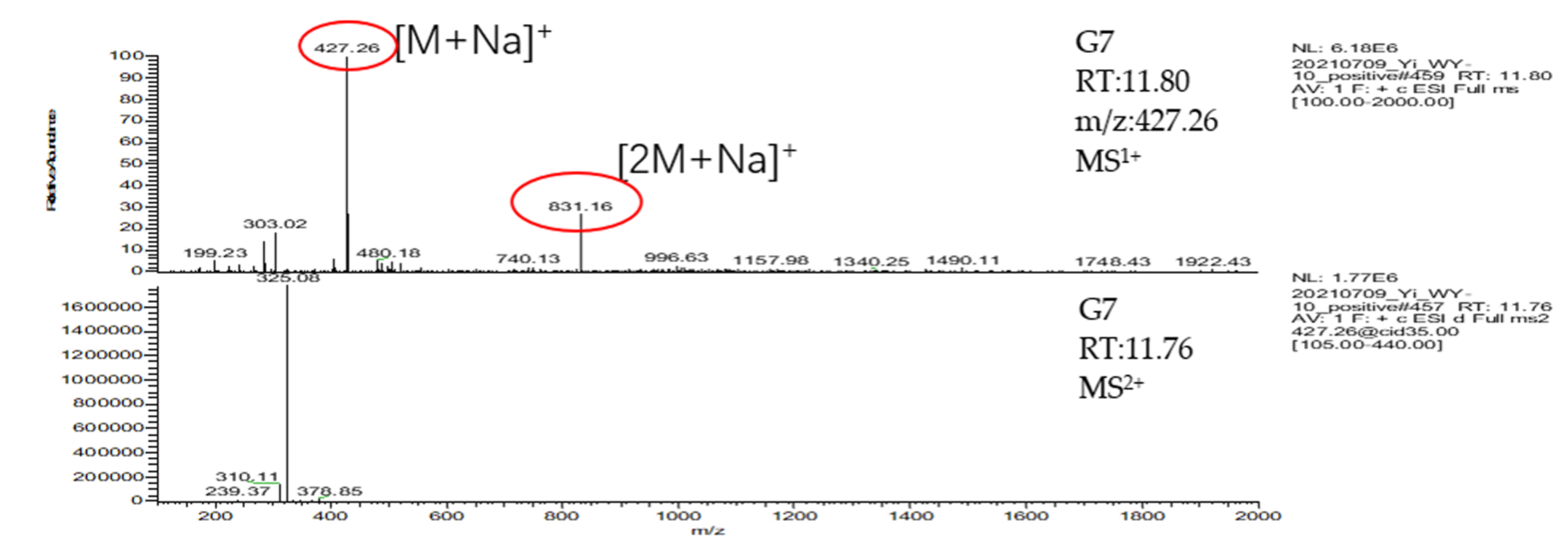

Supplement: Supplementary file 1 [file antibiotics-11-00513-s001.zip › Figure S11.png]

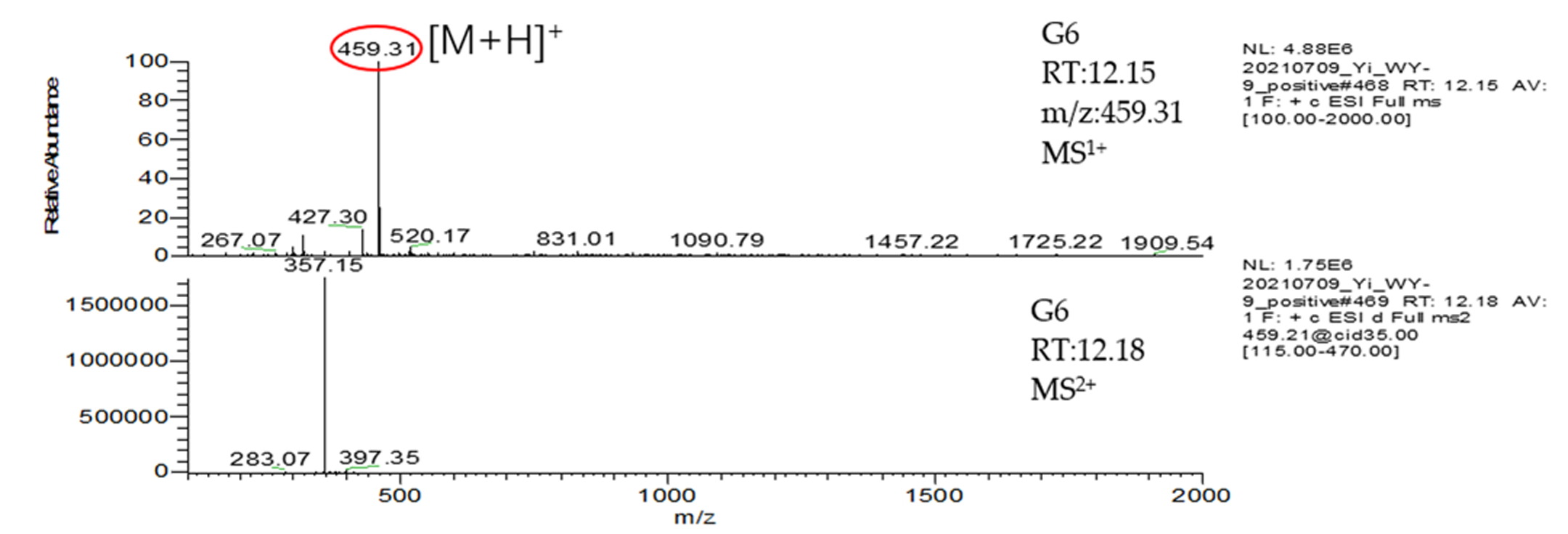

Supplement: Supplementary file 1 [file antibiotics-11-00513-s001.zip › Figure S12.png]

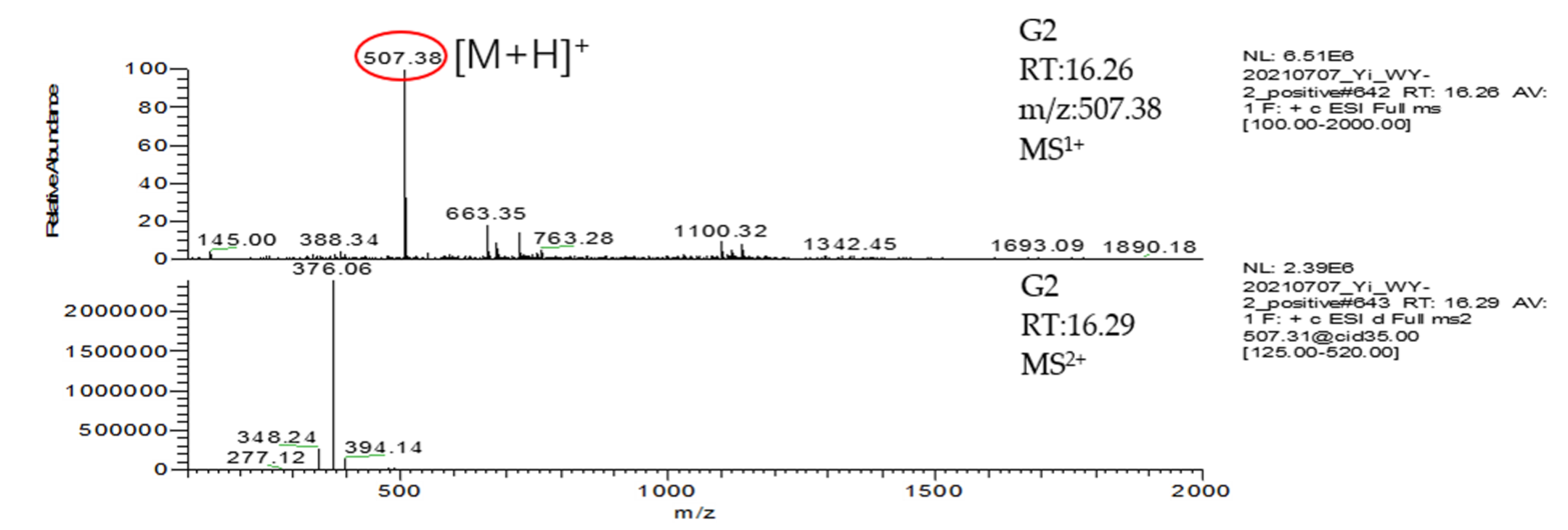

Supplement: Supplementary file 1 [file antibiotics-11-00513-s001.zip › Figure S13.png]

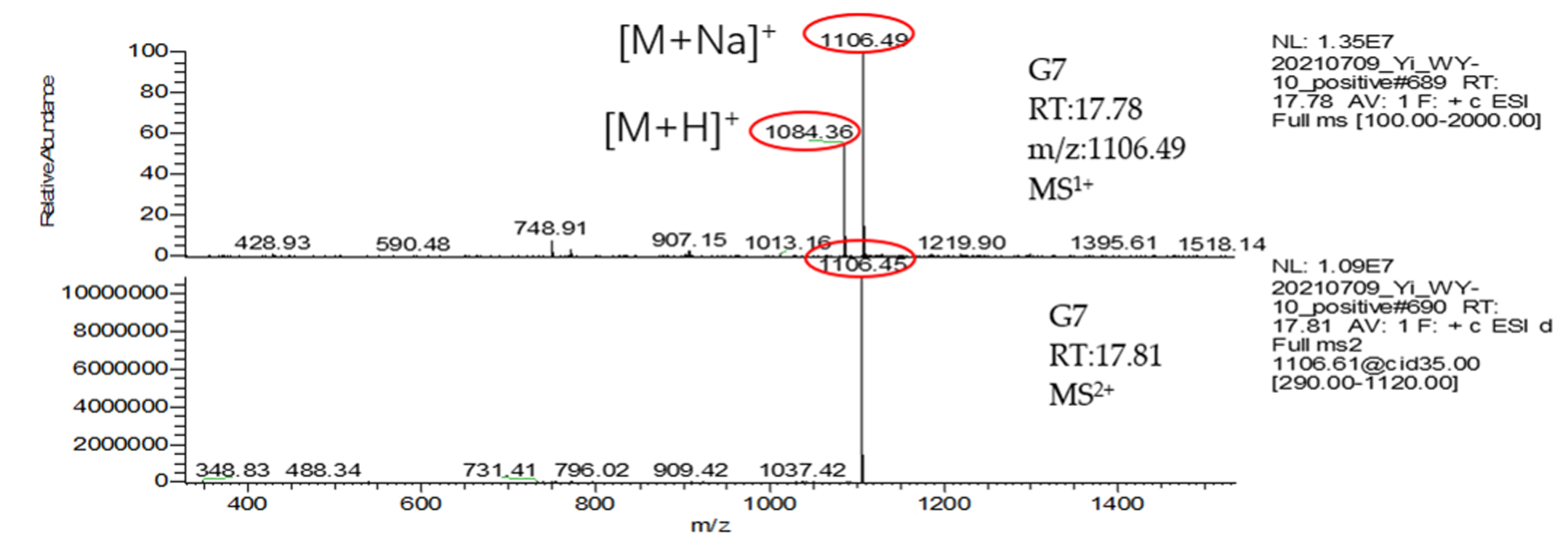

Supplement: Supplementary file 1 [file antibiotics-11-00513-s001.zip › Figure S14.png]

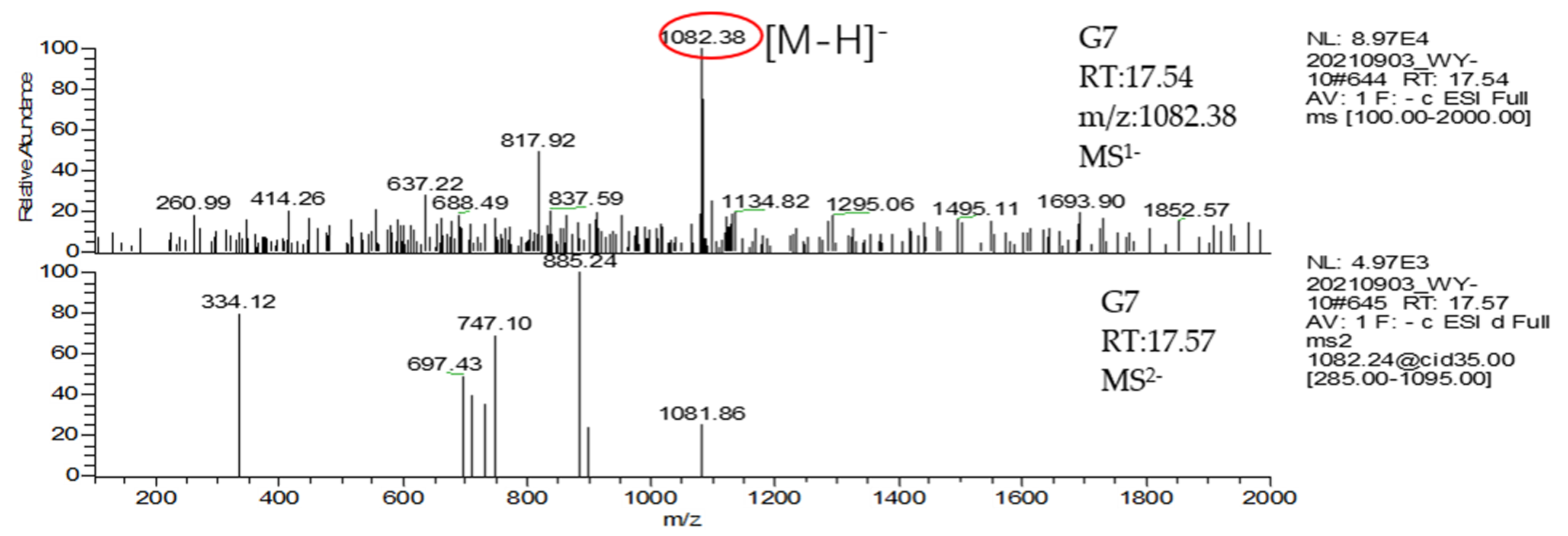

Supplement: Supplementary file 1 [file antibiotics-11-00513-s001.zip › Figure S15.png]

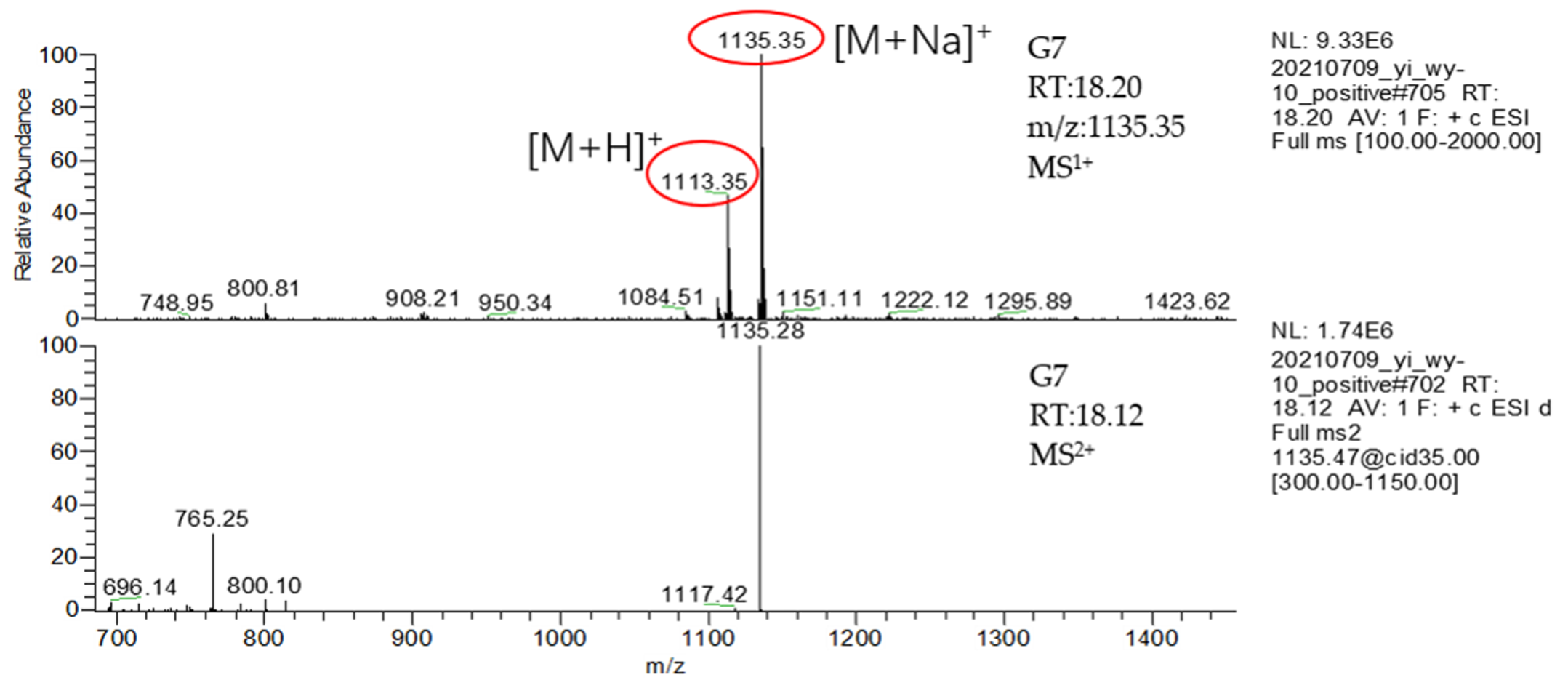

Supplement: Supplementary file 1 [file antibiotics-11-00513-s001.zip › Figure S16.png]

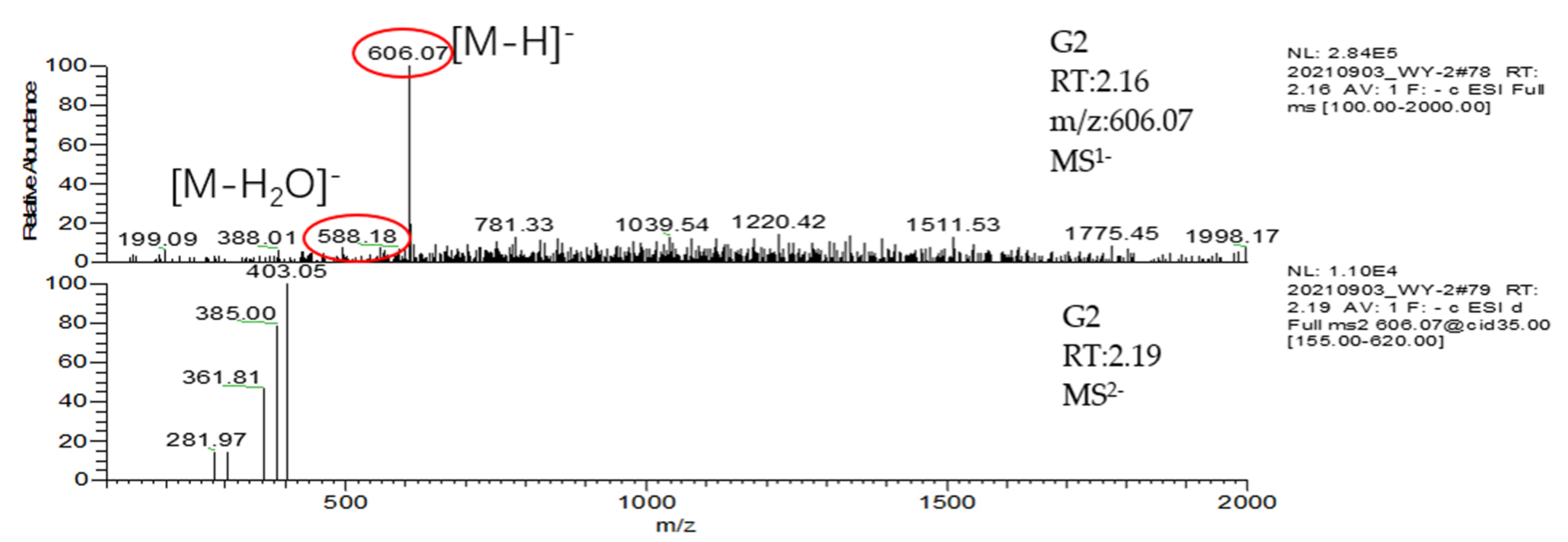

Supplement: Supplementary file 1 [file antibiotics-11-00513-s001.zip › Figure S17.png]

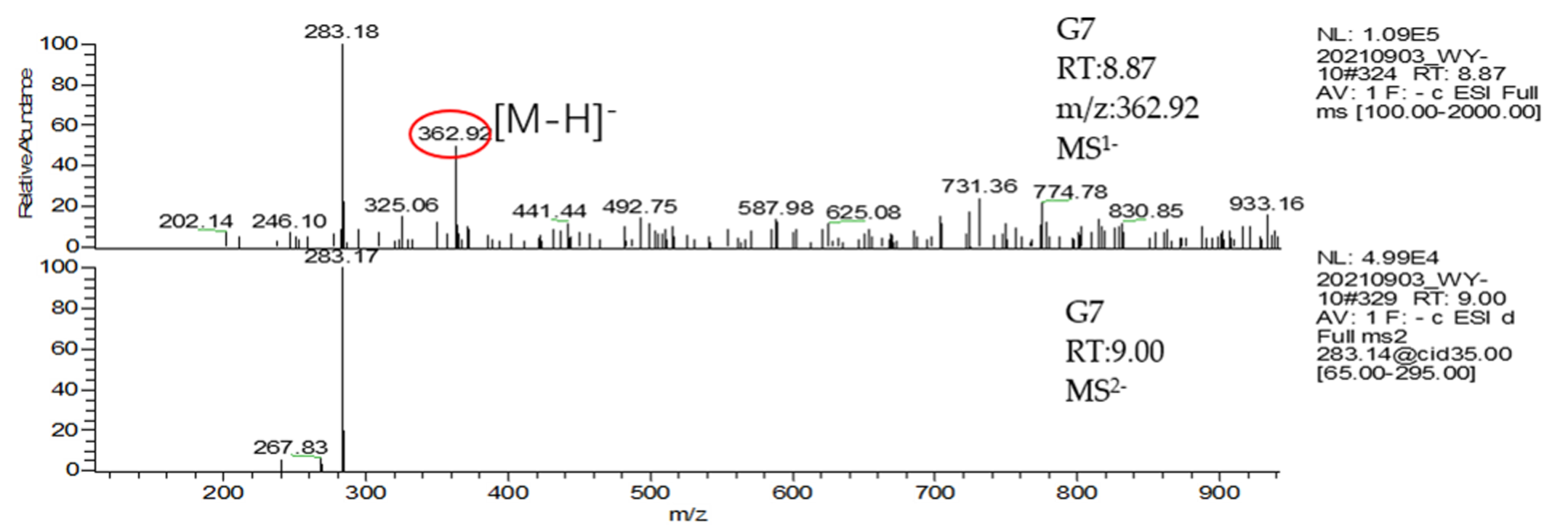

Supplement: Supplementary file 1 [file antibiotics-11-00513-s001.zip › Figure S18.png]

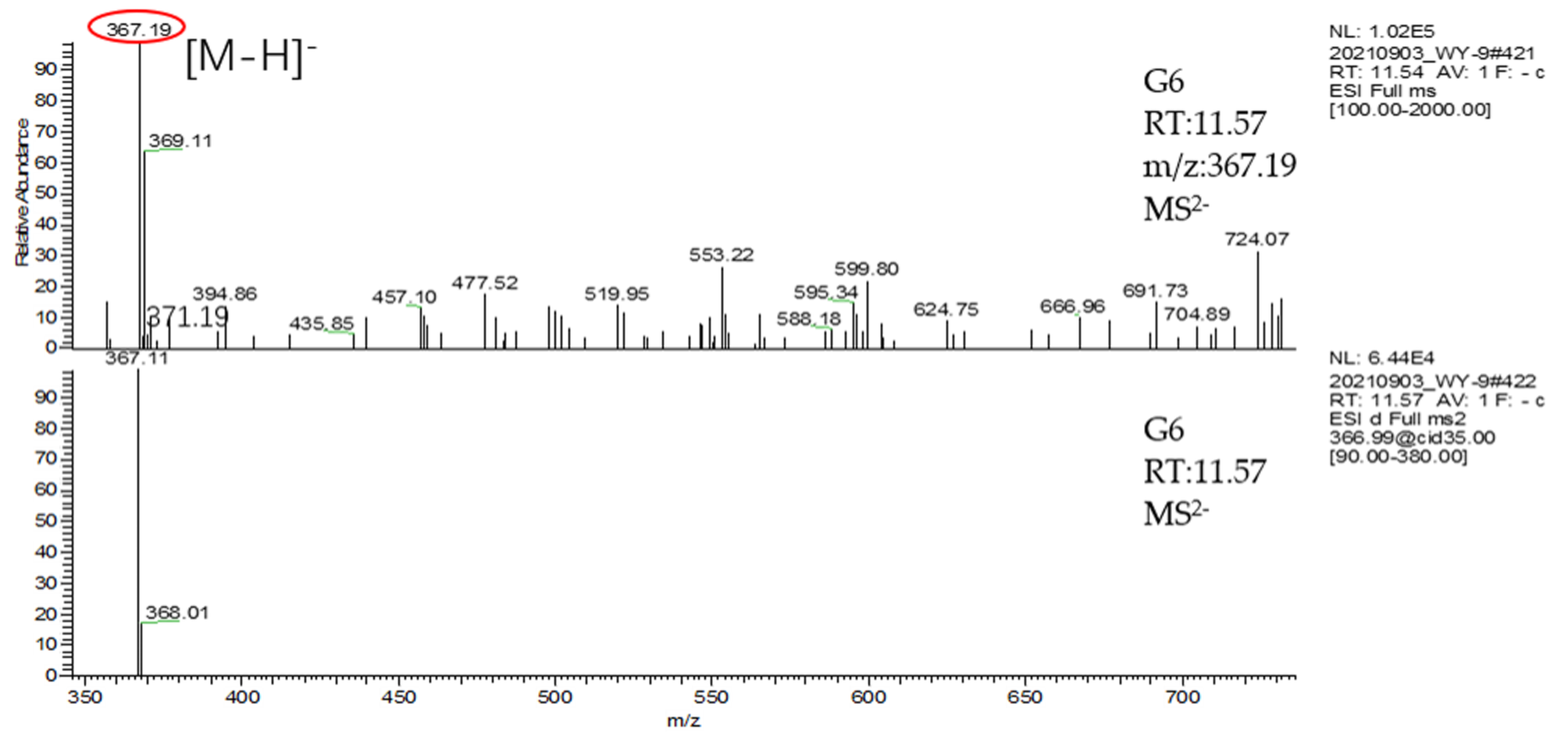

Supplement: Supplementary file 1 [file antibiotics-11-00513-s001.zip › Figure S19.png]

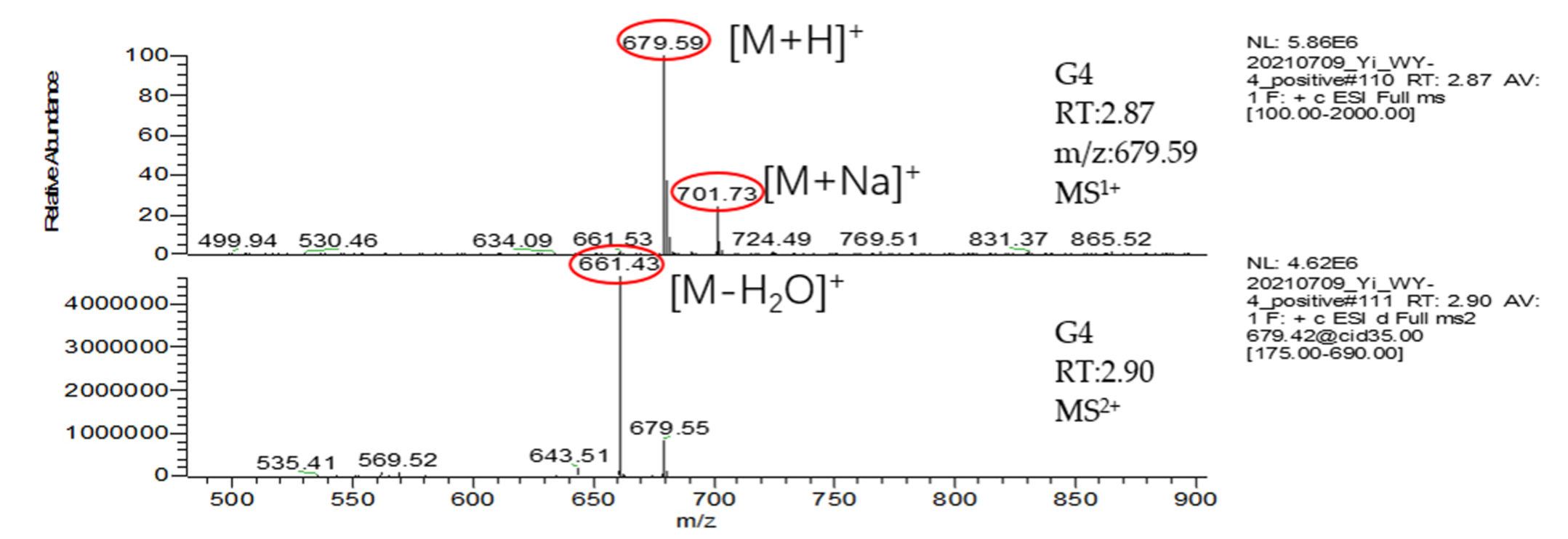

Supplement: Supplementary file 1 [file antibiotics-11-00513-s001.zip › Figure S2.png]

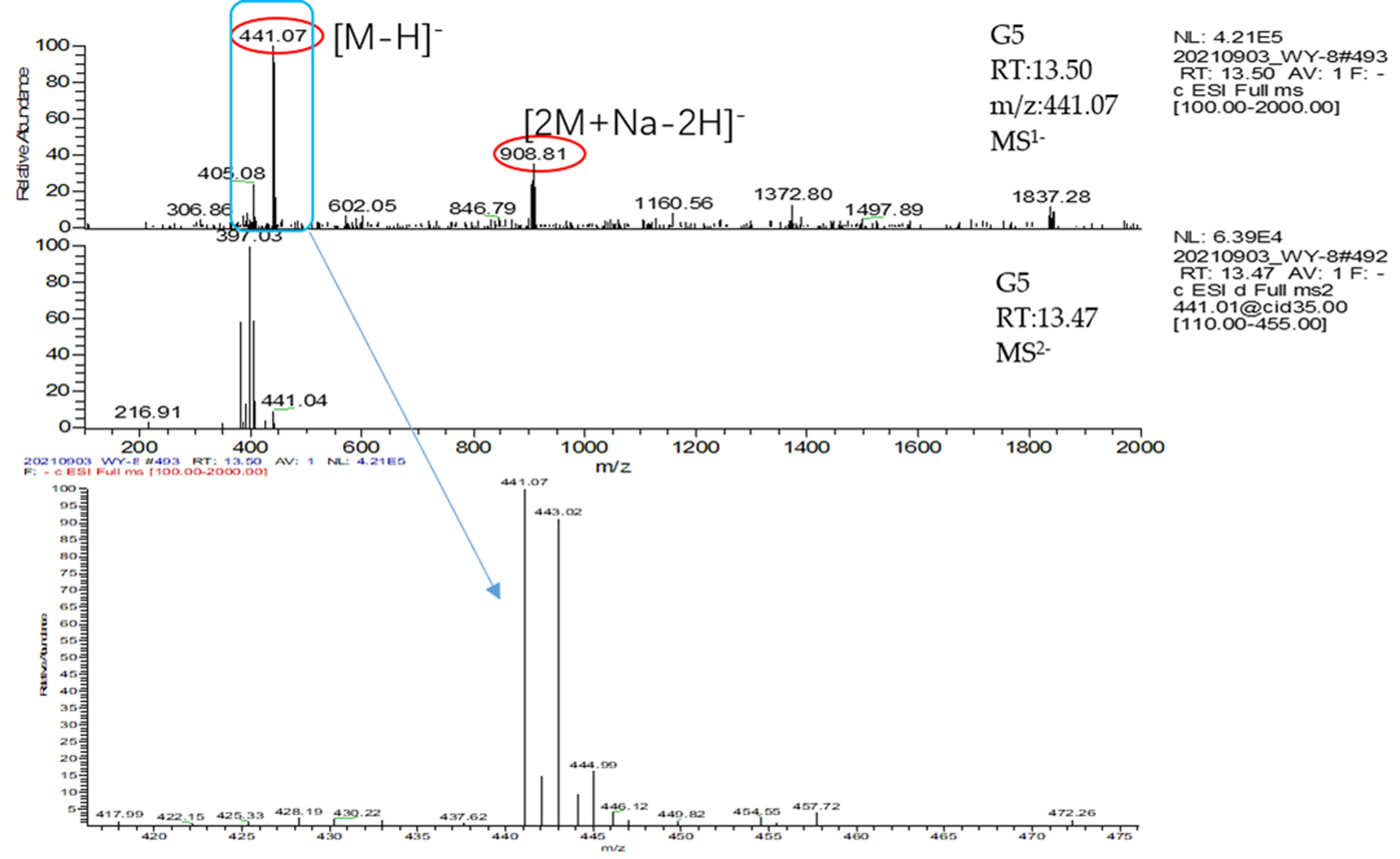

Supplement: Supplementary file 1 [file antibiotics-11-00513-s001.zip › Figure S20.png]

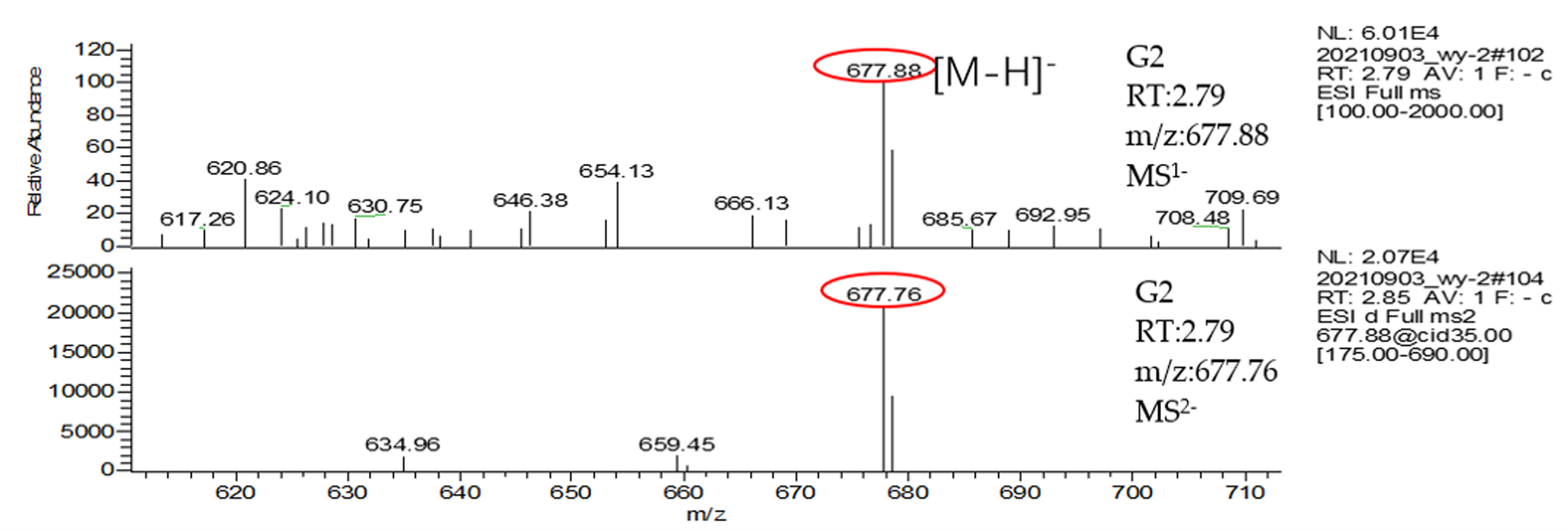

Supplement: Supplementary file 1 [file antibiotics-11-00513-s001.zip › Figure S3.png]

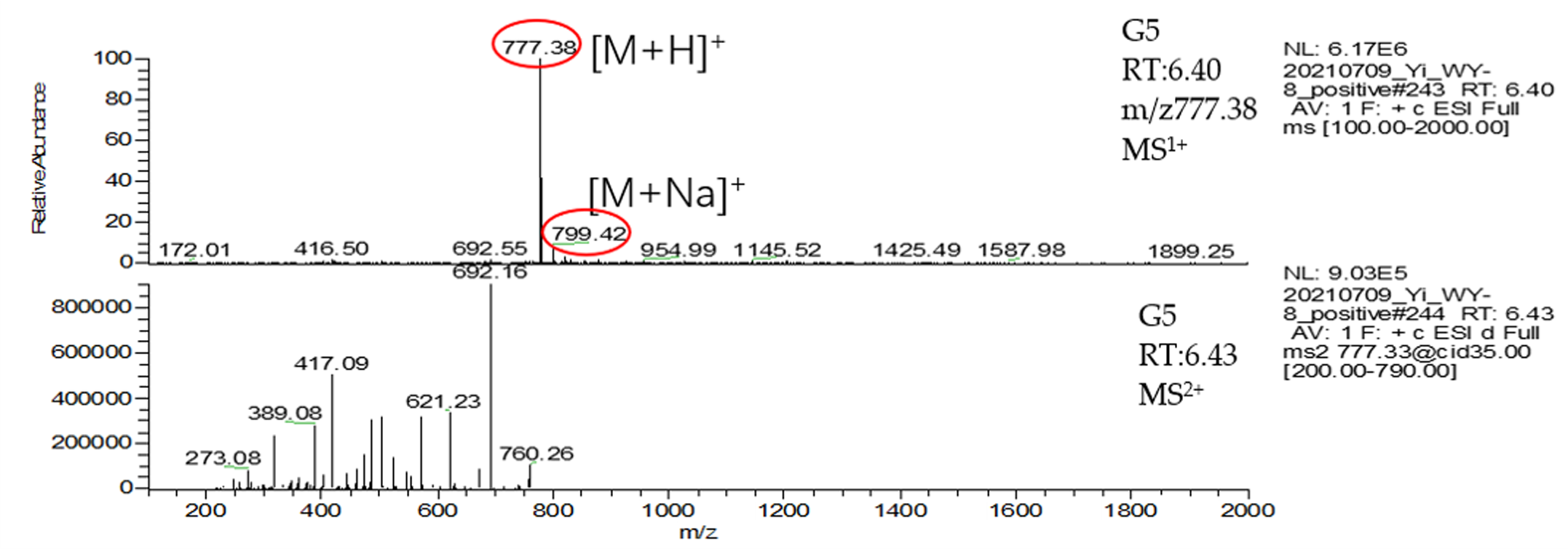

Supplement: Supplementary file 1 [file antibiotics-11-00513-s001.zip › Figure S4.png]

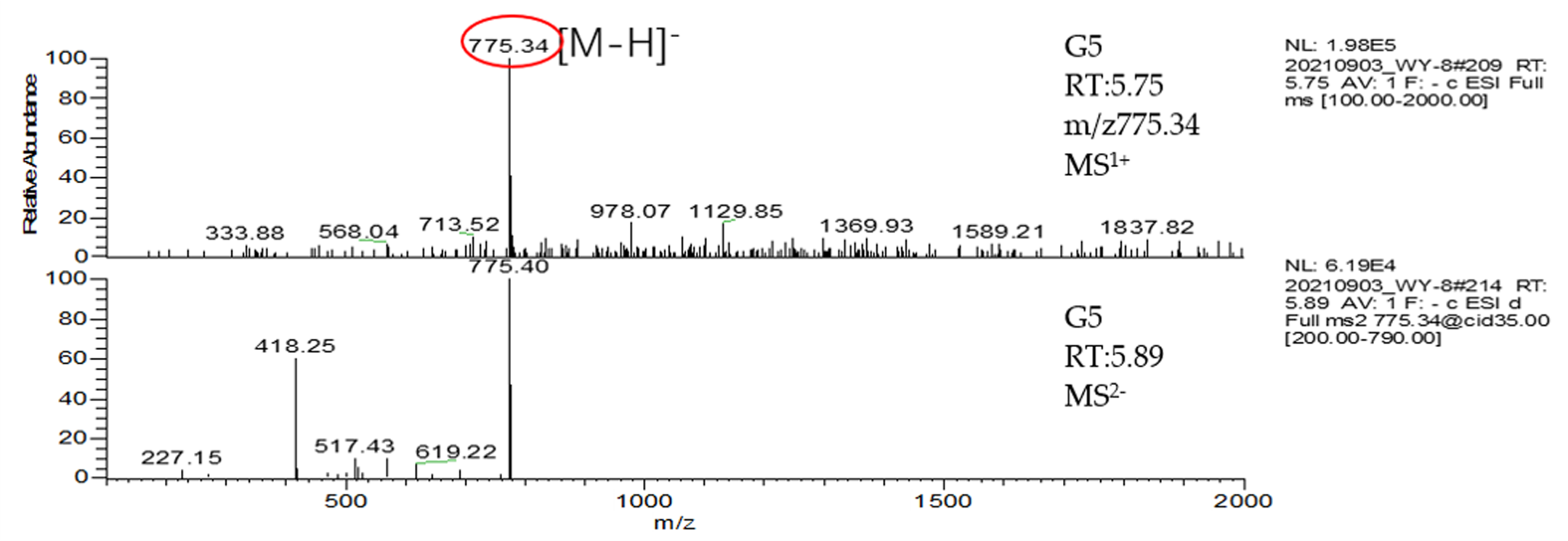

Supplement: Supplementary file 1 [file antibiotics-11-00513-s001.zip › Figure S5.png]

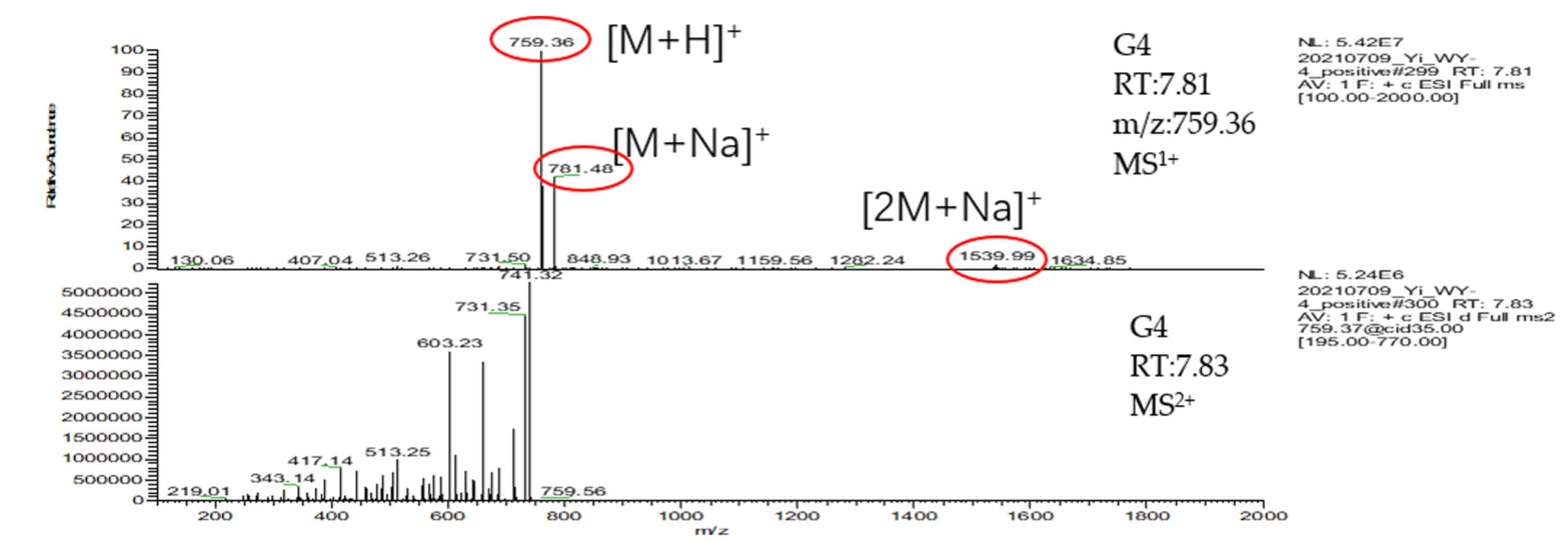

Supplement: Supplementary file 1 [file antibiotics-11-00513-s001.zip › Figure S6.png]

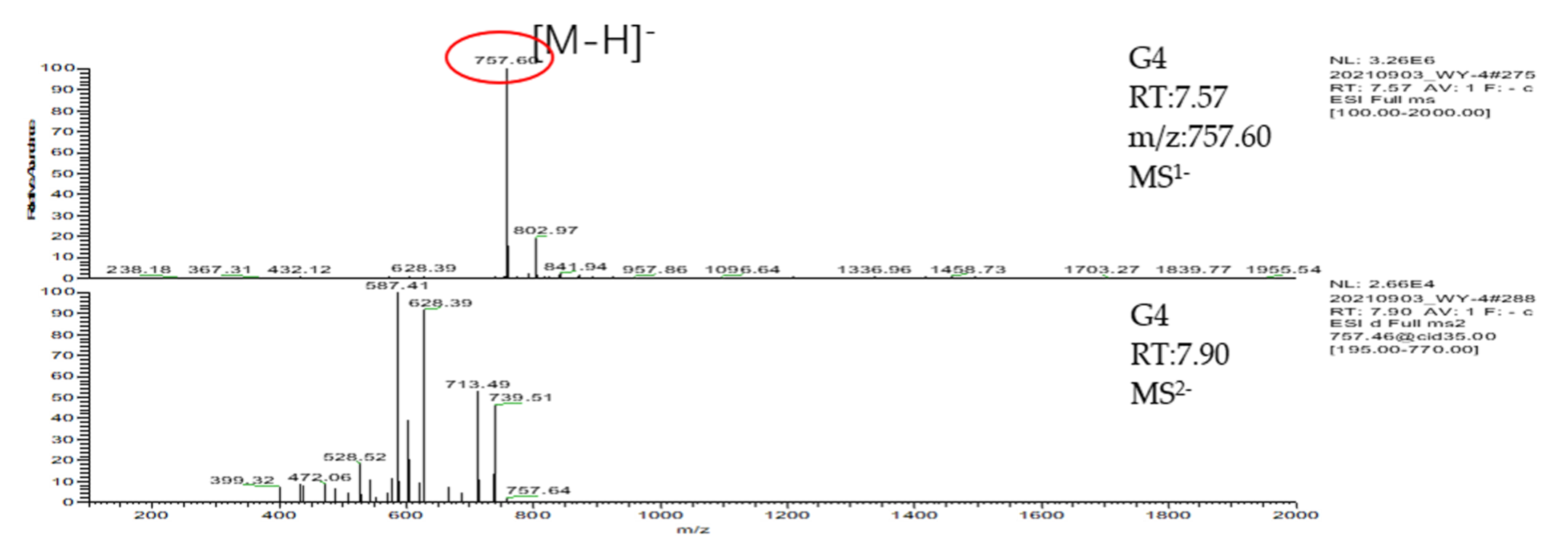

Supplement: Supplementary file 1 [file antibiotics-11-00513-s001.zip › Figure S7.png]

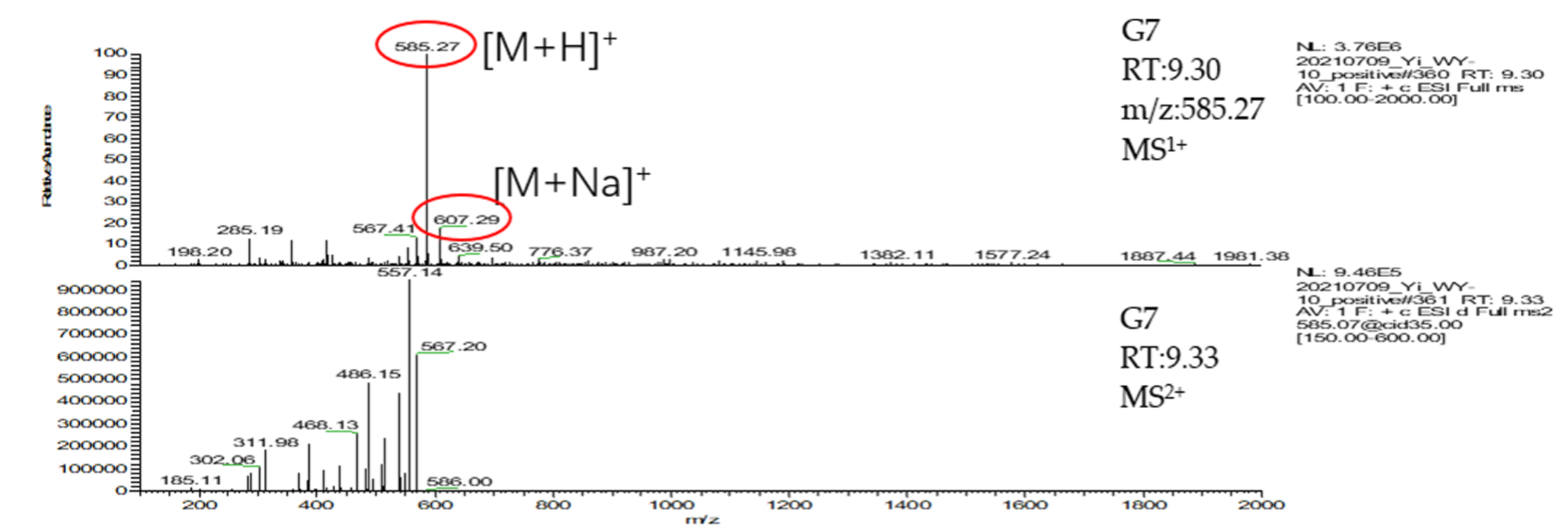

Supplement: Supplementary file 1 [file antibiotics-11-00513-s001.zip › Figure S8.png]

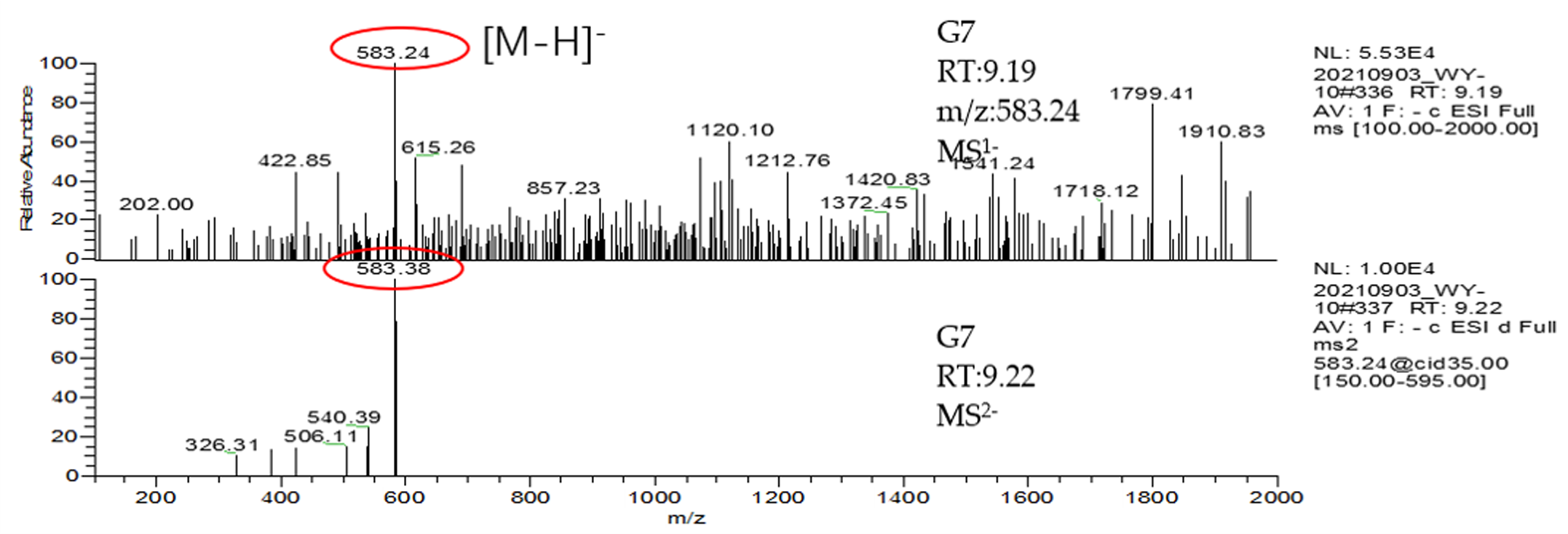

Supplement: Supplementary file 1 [file antibiotics-11-00513-s001.zip › Figure S9.png]
